# What is a reasonable cost to refute a preposterous hypothesis?

**DOI:** 10.1038/sj.bjc.6605523

**Published:** 2010-01-05

**Authors:** A W Castleberry, F W Grannis

**Affiliations:** 1 Department of Surgery, Duke University, Durham, NC, USA; 2 Department of Thoracic Surgery, City of Hope National Medical Center, Duarte, CA, USA

**Sir,**

This letter responds to editorials by SW Duffy and JM Reich commenting on our recent publication ‘Cost of a 5-year lung cancer survivor: symptomatic tumour identification *vs* proactive computed tomography screening’ (Castleberry *et al.*, 2009; Duffy, 2009; Reich, 2009).

Although we appreciate their efforts to analyse our methods and conclusions, we believe that the comments reflect preconceptions and misunderstandings about lung cancer screening. Although our manuscript clearly states that the estimates are based on screening within the context of the International Early Lung Cancer Action Program (IELCAP) Protocol, neither Dr Duffy nor Dr Reich reference this document in their editorials or appear to appreciate its implications (IELCAP, 2009). This is an important omission. Furthermore, Dr Reich's comments contain a number of inaccurate statements regarding the published results of lung cancer screening.

We thank Dr Duffy for his kind remarks on the innovative nature of our method of analysis. To address his comment that survival of screen-detected cases is known to be potentially affected by lead-time and length-time biases, we point out that published IELCAP data demonstrate a flat actuarial survival curve with no drop in survival consistent with delayed lead-time deaths out to 10 years (Henschke *et al*, 2006). IELCAP data also show an incidence of rapidly growing interval lung cancers occurring between annual CT screens of less than 1%, inconsistent with substantial length-time bias (Carter *et al*, 2007).

The primary critique of both editorialists is that our cost estimates may be inaccurate because we did not factor substantial proportions of theoretically non-lethal, overdiagnosed lung cancers in our model. Although Dr Duffy states that ‘it is difficult to avoid the conclusion that there must be some overdiagnosis in CT screening’, he hedges ‘if the hypothesis of a sizable population of indolent lung tumors is correct, this is a fascinating phenomenon, given the very poor prognosis of symptomatic disease’. We believe that his scepticism is judicious.

Reich, however, asks the reader to be sufficiently credulous to accept that there might be sufficiently large numbers of non-lethal screen-detected cancers that a double-arm trial could show a fivefold difference in survival (between symptomatic lung cancers in the US or British national data (8–15%) and 80% actuarial 10-year survival in the IELCAP study) and yet no reduction in lung cancer-specific mortality.

To support this hypothesis, Reich estimates that the incidence of overdiagnosed lung cancer is around 50%. In fact, to explain this markedly improved survival without reduced deaths, more than 75% of CT-screen detected lung cancers would have to be non-lethal.

In the case of prostate cancer, for which the existence of a substantial number of very slow-growing cancers has been well established, a recent publication from the National Cancer Institute (NCI) estimates that the incidence of overdiagnosis is approximately 23% (Welch and Albertsen, 2009). Reich therefore asks the reader to accept that the incidence of overdiagnosed lung cancer could double that of prostate cancer. But there is no reliable direct evidence to document any substantial lung cancer overdiagnosis. Three 1986 publications from Alvan Feinstein describing ‘postmortem-surprise lung cancers’ were funded by the notorious Council for Tobacco Research and are often cited in defence against medical monitoring lawsuits (McFarlane *et al*, 1986a, 1986b, 1987). Symptom-detected but untreated stage I patients die, almost always, within 5 years (Raz *et al*, 2007). The same is true of untreated patients detected by screening roentgenograms and CT scans (Sobue *et al*, 1992).

Reich's hypothesis would be cause for amusement if not for the cost. We refer not to the $200 million price tag of the NCI's National Lung Screen Trial, but to the unnecessary suffering and death of thousands of individuals from lung cancer who might have been salvaged by screening, between today and the publication of prospective randomised trials. Is this a reasonable and humane cost to refute a preposterous hypothesis?

We suspect that a mindset that would consider such cost justifiable is what led Arthur Golleb to characterise epidemiology as ‘the practice of medicine without the tears’.
